# 3D Models as a Tool to Assess the Anti-Tumor Efficacy of Therapeutic Antibodies: Advantages and Limitations

**DOI:** 10.3390/antib11030046

**Published:** 2022-07-08

**Authors:** Virginia Guzzeloni, Lorenzo Veschini, Federica Pedica, Elisabetta Ferrero, Marina Ferrarini

**Affiliations:** 1 B-Cell Neoplasia Unit, Division of Experimental Oncology, IRCCS Ospedale San Raffaele, 20132 Milan, Italy; guzzeloni.virginia@hsr.it (V.G.); ferrero.elisabetta@hsr.it (E.F.); 2 Academic Centre of Reconstructive Science, Faculty of Dentistry Oral & Craniofacial Sciences, King’s College London, Guy’s Hospital, London SE1 9RT, UK; lorenzo.1.veschini@kcl.ac.uk; 3 Pathology Unit, IRCCS Ospedale San Raffaele, 20132 Milan, Italy; pedica.federica@hsr.it

**Keywords:** therapeutic monoclonal antibodies, 3D models, tumor microenvironment, drug response

## Abstract

Therapeutic monoclonal antibodies (mAbs) are an emerging and very active frontier in clinical oncology, with hundred molecules currently in use or being tested. These treatments have already revolutionized clinical outcomes in both solid and hematological malignancies. However, identifying patients who are most likely to benefit from mAbs treatment is currently challenging and limiting the impact of such therapies. To overcome this issue, and to fulfill the expectations of mAbs therapies, it is urgently required to develop proper culture models capable of faithfully reproducing the interactions between tumor and its surrounding native microenvironment (TME). Three-dimensional (3D) models which allow the assessment of the impact of drugs on tumors within its TME in a patient-specific context are promising avenues to progressively fill the gap between conventional 2D cultures and animal models, substantially contributing to the achievement of personalized medicine. This review aims to give a brief overview of the currently available 3D models, together with their specific exploitation for therapeutic mAbs testing, underlying advantages and current limitations to a broader use in preclinical oncology.

## 1. Introduction

Immunotherapy is the newest and fastest growing branch in clinical oncology [1]. Among several approaches, therapeutic antibodies have already revolutionized the treatment of both solid and hematological malignancies, with dramatic improvement of clinical outcomes [2,3,4]. This substantial success has fueled the development of a growing number of immunotherapy agents and the design of novel combination therapies. However, it is often challenging to identify patients who are likely to benefit from therapy and to design tailored therapeutic regimens. As a result, mAbs therapies have not completely fulfilled all the expectations, due to limited efficacy and/or significant toxicity [2,3]. Additional strategies to generate safer and more effective anti-tumor immune responses are urgently needed and actively pursued. Cancer drug research and development progresses slowly, with an estimated time frame of 10–12 years for new cancer drug developments, and less than 5% probability for candidate drugs entering clinical trials to receive the US Food and Drug Administration (FDA) approval [5,6,7]. A major challenge to wider application of mAb-based therapies in clinical oncology is represented by the lack of reliable biomarkers to accurately identify patients who most likely will benefit from treatment with a given antibody [3]. In this regard, it is increasingly recognized that advanced in vitro models for cancer drugs discovery and therapeutic assessment can help to expedite the transition from bench to the bedside [8]. 

Novel three-dimensional (3D) in vitro models recapitulating cancer biology within its microenvironment have been established as significant improvement in comparison to less biomimetic 2D culture systems. Such 3D models are providing more predictive systems for personalized medicine, therapeutic drug screening and preclinical research [8].

Here, we review the most relevant 3D cancer models developed to assess the impact of anti-tumor therapies, with a special focus on therapeutic mAbs. We highlight potential advantages over conventional methods for drug testing and overview present limitations to a broader use in clinical oncology.

## 2. Therapeutic mAbs in Cancer Treatment

The discovery and development in 1975 of the hybridoma technology by George Kohler and Cesar Milstein [9] paved the way to targeted therapies, making mAbs a fundamental tool for biomedical science. mAbs have profoundly influenced human therapeutics in a wide range of disorders, encompassing autoimmune, infectious, cardio-vascular, neurological diseases and many types of cancer [2,3,4]. Moreover, during the current COVID-19 pandemic, a variety of prophylactic and therapeutic treatments are being developed to fight the virus, including virus-neutralizing mAbs [10]. In the field of cancer treatment, since the approval in 1997 of the first mAb (rituximab) by the US FDA, an ever-increasing number of mAbs has been developed [11], making them a major therapeutic option for many cancer types in the current oncologic practice. Specifically, the “Antibodies to Watch” article series, which reviews on annual basis approvals of novel therapeutic mAbs and candidate products, listed, as of November 2021, more than 130 mAbs in the US and EU, nearly half being treatments for cancer [12]. 

**Figure 1:**
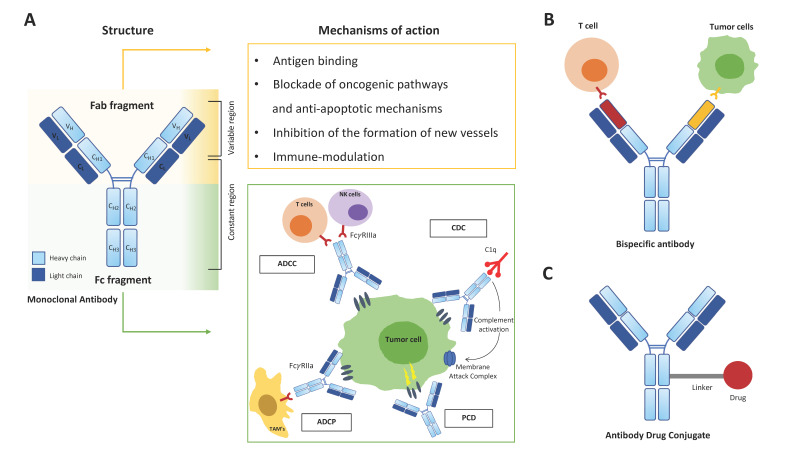
Structure and mechanisms of action of therapeutic mAbs:(**A**) mAbs consist of two identical Heavy chains (light blue) and two identical Light chains (blue) connected by disulphide bonds. The antigen-binding fragment (Fab) is composed of one constant and one variable domain of each of the Heavy and the Light chain; the variable domains contain the antigen-binding site, which determines antigen recognition and the mechanisms of action of the mAb. The constant (Fc) portion of an antibody mediates the antibody’s immunological effects, including Antibody-Dependent Cell-mediated Cytotoxicity (ADCC), Programmed Cell Death (PCD), Antibody-Dependent Cell Phagocytosis (ADCP) and Complement-Dependent Cytotoxicity (CDC); (**B**) schematic representation of an IgG-like bispecific mAb redirecting a T effector cell towards its tumor target cell; (**C**) Antibody Drug Conjugate consisting in a mAb linked to a cytotoxic drug (payload).

The generation of therapeutic mAbs started with the synthesis of mAbs of murine origin (-omab), followed by the fast development of chimeric mAbs, containing variable regions from mice and the remainder from human sources (-ximab), humanized mAbs, with only a mouse-derived antigen-binding fragment (-zumab), up to fully human mAbs (-umab), which represent the gold standard [13]. Nowadays, mAbs are being developed to target different molecules with different mechanisms of action. Ideal target molecules are surface antigens that are highly expressed on tumor cells and not (or at low density) on normal tissues, thus promoting on-target activity while limiting off-target toxicity. Prototypical mAbs targeting antigens expressed by hematological tumors are the anti-CD20 mAb rituximab, currently used for treatment of B-cell lymphomas [14,15,16] as well as for B-cell depletion in severe autoimmune and neurological diseases [17,18,19], and the anti-CD38 mAbs daratumumab and isatuximab, in use for treatment of Multiple Myeloma (MM) and light chain amyloidosis [20,21,22]. The overall anti-tumor effects of mAbs are driven both by their antigen-binding regions and by the properties of their Fc domains, which confer the capability to bind to components of the complement cascade and to Fc receptors expressed by neutrophils, NK cells, monocytes and dendritic cells [23,24]. Thus, in addition to their antigen specificity, mAbs differ in their efficiency in mediating Antibody-Dependent Cell-mediated Cytotoxicity (ADCC), Programmed Cell Death (PCD), Antibody-Dependent Cell Phagocytosis (ADCP) and Complement-Dependent Cytotoxicity (CDC) of tumor cells, which varies in the different subtypes of immunoglobulins (Igs) [23,24,25] (Figure 1A). ADCC is the antibody-induced lysis of a target cell by activated effector cells, mainly NK cells. mAbs binding to target cells through their variable regions, and to FcγRIIIa expressed on immune effectors through their Fc region, results in the recruitment of adapter proteins and activation of the effector cells, followed by release of lytic factors and target destruction [23]. ADCP is a related mechanism mediated by FcγRIIa-activated macrophages leading to augmented phagocytosis of opsonized targets [23]. PCD represents a caspase-independent, non-apoptotic cell death induced by upregulation of pro-apoptotic factors and downregulation of anti-apoptotic factors upon FcγR-mediated mAbs cross-linking at the target cell surface [25]. Finally, CDC occurs when mAbs bound to target cells recruit the complement subunit C1q, leading to activation of the classical pathway of the complement cascade and ultimately to formation of the membrane attack complex and cell lysis [23] (Figure 1A).

With regard to antigen binding, current cancer targeted mAbs-mediated immunotherapies aim to achieve different goals including (i) blockade of oncogenic pathways regulating tumor cell growth/survival and apoptosis, (ii) inhibition of the formation of new vessels (neo-angiogenesis) and (iii) immune-modulation (Figure 1A). 

(i) *Blockade of oncogenic pathways*. Tumor initiation and progression is usually linked to the acquisition of driver mutations that over activate proto-oncogenes and/or inactivate tumor suppressor genes governing cellular programs involved in tumor growth/survival, cell death and metabolism [26,27]. Tumor viability and growth can then be perturbed with targeted mAbs capable of modulating specific intracellular signaling pathways by targeting corresponding membrane receptors. Examples include Epidermal Growth Factor Receptor (EGFR), which upon ligand binding promotes cell proliferation, migration and invasion via activation of the Mitogen-Activated Protein Kinase (MAPK) and AKT pathways [28]. Tumor cells overexpressing EGFR, including metastatic colorectal cancer (CRC) and head and neck cancer, may undergo constitutive activation that can be targeted by mAbs such as cetuximab, which block ligand binding and/or receptor dimerization, resulting in cell cycle arrest and apoptosis. Human epidermal growth factor receptor 2 (HER2) is an established therapeutic target in a large subset of women with breast cancer and is also overexpressed in subsets of patients with other solid tumors [29]. Homo- and hetero-dimerization of HER2 with other members of the EGFR family, typically due to its overexpression, initiates a variety of signaling pathways implicated in cellular proliferation and tumorigenesis [29]. Antibodies targeting HER2 (trastuzumab and pertuzumab) achieve their signaling perturbation effects by inhibiting receptor homo- and hetero- dimerization and internalization, rather than by blocking ligand binding [30,31]. It is increasingly recognized that cancer initiation and development promote a wide array of dynamic structural alterations inside host tissues aimed at assisting tumor cell proliferation and survival and conferring drug resistance [32]. The resulting abnormal milieu, also known as the tumor microenvironment (TME), comprises several non-malignant cellular populations, extra-cellular matrix (ECM) components, and other secreted molecules, such as growth factors, cytokines and chemokines [32]. In turn, essential elements of the tumor stroma, such as immune responses, inflammation, angiogenesis, metabolism and hypoxia, deeply influences tumor progression and metastasis and have been widely investigated as potential therapeutic targets [32]. The most advanced strategies, which either have been approved or are currently under clinical evaluation, mainly focus on the targeting of tumor vasculature and of immune cells, as discussed hereafter.

(ii) *Inhibition of the formation of new vessels*. Cancer cell growth and metastasis depend on tumor capability to develop an adequate blood supply by intra-tumor neo-angiogenesis [33]. Over the past decades, this process has been progressively elucidated, unveiling novel targets of therapeutic intervention in the fight against cancer [34,35,36]. Tumor angiogenesis is promoted by pro-angiogenic factors, including Vascular Endothelial Growth Factor (VEGF), Platelet Derived Growth Factor (PDGF), Fibroblast Growth Factor (FGF) and members of the Angiopoietins family [34,35,36,37]. Such molecules can be produced by both stromal and tumor cells in the TME inducing the “angiogenic switch”, leading to the neo-formation of intra-tumoral blood vessels [34,35,36,37]. Of note, compared with healthy tissues, tumor neo-vasculature is often structurally aberrant and dysfunctional, resulting in inefficient oxygen delivery, intra-tumor hypoxia, dysregulated inflammatory response, and impaired delivery and distribution of therapeutic agents, overall concurring to increased cancer aggression [38]. Traditional anti-angiogenic strategies are aimed at reducing the vascular supply with the goal of starving the tumor [34,35,36]. Both VEGF and its receptor (VEGFR-2) have been targets of anti-VEGF-based antiangiogenic therapies, three of which are licensed for clinical use. Bevacizumab, the first vascular-targeting mAb approved by the FDA that specifically binds to VEGF-A and prevents it from binding to its receptor [34,35,36], is routinely used in the treatment of several solid tumors, including metastatic CRC and metastatic renal cell carcinoma (RCC) [34,35,36]. An alternative approach to blocking VEGF-driven angiogenesis relies in the use of small molecule tyrosine-kinase inhibitors (TKI) that target VEGF receptors. Two such inhibitors, sorafenib and sunitinib, have been licensed by the FDA for treatment of solid tumors (unresectable hepatocellular carcinoma-HCC, advanced RCC, gastrointestinal stromal tumors) [34,35,36]; several other inhibitors are under evaluation in clinical trials testing [34]. Unfortunately, despite very promising results in preclinical evaluation, anti-angiogenic treatments as monotherapy displayed limited efficacy in clinical trials [39]. Patients develop resistance over time or are primarily refractory to anti-angiogenic therapies, due to redundant pro-angiogenic factors and alternative ways to form new vessels, which do not exploit the sprouting from existing vessels, such as recruitment of bone marrow-derived vascular progenitor cells that differentiate toward EC (vasculogenesis) [40]. Accordingly, an emerging strategy exploiting anti-angiogenic agents aims at normalization of tumor vascular abnormalities as a pre-requisite for efficient delivery of combination immune- or chemo-therapies [35,38,41,42]. Structural and functional vessel normalization can be induced rapidly and maintained for a transient time window through the careful dosing of anti-angiogenic therapies [35]. This strategy results in better tumor perfusion and multiple benefits such as increased efficacy of chemotherapy due to enhanced intra-tumor drug delivery and immunotherapy potentiation by guiding immune effector cells infiltration overall improving patient outcome [41,42]. However, given the high variability in patients’ response to anti-angiogenic drugs, it is currently challenging to determine the proper dose of treatment as well as the duration of the therapeutic window, in which vessel normalization is promoted, and combination therapy can be successfully administered. To estimate the optimal time window for therapeutic intervention, availability of reliable predictive biomarkers and of detailed vascular visualization and quantification are fundamental [43].

(iii) *Immune-modulation*. Finally, therapeutic mAbs have been developed with the aim of interfering with cancer-induced immune response. Advances in tumor biology have clearly demonstrated the occurrence of a tight and double-edged interplay between malignant cells and the immune system. On one hand, the immune system can recognize and eliminate transformed cells (“immune surveillance”); on the other hand, the mutual interactions and crosstalk between immune cells and cancer cells within their microenvironment are implicated in cancer development and progression (“immune-editing”) [44]. Accordingly, both resistance to immune attack and pro-tumoral inflammation have been incorporated into the major hallmarks of cancer [45]. Thanks to the progressive identification of mechanisms governing tumor immune control and evasion, the immune system is now considered a promising target for novel and more effective therapeutic strategies.

Therapeutic approaches based on immune checkpoint inhibitors (ICI) have dramatically transformed the management of advanced-stage melanoma, RCC and several other types of cancer [46,47,48], as also recognized by the 2018 Nobel Prize in Physiology and Medicine awarded to James Allison and Tasuku Honjo for their development of cancer therapy through the inhibition of negative immune regulation [49]. Immune checkpoints are inhibitory receptors that convey negative signals to immune cells [50]. They include Cytotoxic T lymphocyte antigen-4 (CTLA-4), which restrains antigen-driven activation of naive T cells by competitive binding to the costimulatory receptors B7.1 (CD80) and B7.2 (CD86) on antigen-presenting cells (APCs), and Programmed cell death-1 (PD-1), which, upon engagement by its ligand (PD-L1, Programmed cell death ligand 1), inhibits the activity of effector T cells [50]. Both CTLA-4 and the PD-1/PD-L1 axis operate under physiologic conditions to attenuate excessive immune responses and prevent autoimmunity; however, they can be co-opted by the tumor to escape from recognition and destruction by the immune system [46,47]. Accordingly, disposal of inhibitory signals of T-cell activation through immune checkpoint blockade enables tumor-reactive T cells to mount an effective anti-tumor response [46,47]. So far, different mAbs targeting CTLA-4 (ipilimumab), PD1 (nivolumab and pembrolizumab) and PDL-1 (atezolizumab, avelumab, and durvalumab) are FDA approved for a broad range of cancers [46,47], and the therapeutic potential of many of other targets, including Lymphocyte Activating 3 (LAG3), T cell immunoglobulin and mucin-domain containing-3 (TIM3), TIGIT, is now being investigated preclinically and in clinical trials [51].

Chronic inflammation has long been recognized as one of the major drivers for both carcinogenesis and tumor progression in several cancer cell types [52]. Cytokines derived from many cellular sources are responsible for key cell–cell interactions inside the TME, and significantly contribute to tumor growth along with co-evolving immune responses. Among pro-tumoral cytokines, Interleukin (IL)-1 not only promotes inflammation-induced carcinogenesis but also contributes to tumor invasiveness and angiogenesis [53,54,55]. IL-6 amplifies pro-carcinogenic chronic inflammation and drives tumor-intrinsic mechanisms of cancer progression, including tumor growth and apoptosis resistance, metabolic rewiring, angiogenesis, and metastasis [56,57]. The potential of cytokines as therapeutic targets is highlighted by the increasing number of clinical trials currently under way [44]. For example, IL-1 neutralization through the anti-IL-1ß antibody canakinumab or the IL-1 receptor antagonist anakinra is under investigation in ongoing clinical trials in multiple cancer types, both as monotherapy and in combination therapy [44,55], while IL-6 targeting with the anti-IL-6 Receptor antibody tocilizumab is under evaluation in combination with ICI with conflicting results [44]. Finally, given the multifaceted effects of Tumor Necrosis Factor (TNF)-α in inflammation and anti-tumor immunity, encompassing cytotoxic effects on cancer cells, but also autocrine/paracrine tumor-promoting outcomes [58], cytokine neutralization through the anti-TNF-α mAbs infliximab and adalimumab or the recombinant TNF-R etanercept has restricted application in cancer therapy, mainly related to treatment of cancer cachexia [59] and of steroid resistant immune-related adverse events (irAEs) following ICI administration [60].

Antibody structural derivatives also contribute to the growing clinical immunotherapy arsenal. Among them, bispecific antibodies (bsAb) (Figure 1B), which comprise a large family of molecules designed to recognize two different epitopes/antigens, hold the potential for novel functionalities, dependent on the physical linkage of the two specificities and not present in mixtures of the parental antibodies [61]. Catumaxomab, the first bispecific T cell engager able to redirect killer T cells directly to tumor cells via two engineered antigen-binding sites (a T lymphocyte antigen CD3 × epithelial cell adhesion molecule -EpCAM), was clinically approved in 2009 for the intraperitoneal treatment of malignant ascites [62]. Since then, progressive advances in the fields of antibody biology and engineering have led to more than 100 bsAb formats, with 2 bsAbs marketed and over 85 in clinical development [61]. Beside immune effector cell redirection towards both solid and hematological malignancies, bsAbs are designed to perform different mechanisms, including double IC inhibition and dual signaling inhibition [61,62]. Examples include PD-1xCTLA-4, PD-1xLAG3 and PD-1xTIM3 bsAbs (currently under evaluation for treatment of solid and hematological malignancies), and EGFR × MET bsAbs, blocking EGFR and MET signaling through inhibition of ligand-induced activation and receptor degradation, for treatment of non-small-cell lung cancer [61]. BsAbs come in different formats, ranging from relatively small proteins, consisting of two linked antigen-binding fragments, to large IgG-like molecules with additional domains attached. Various platforms, including multi-specific antibody-based formats containing three or more antigen-binding sites, are currently in development and clinical trials for multiple malignancies [61,62,63,64].

Anti-tumor agents can also be ferried by antibodies to tumor cells and exert their effects with decreased collateral damage to healthy tissues. Antibody-drug conjugates (ADC) (Figure 1C) are mAbs linked to cytotoxic drugs (payloads) and are designed to improve the therapeutic index of antineoplastic agents by limiting their delivery specifically to cells that express the target antigen [65]. Again, the ideal target is represented by a cell-surface protein that is highly preferentially expressed on tumor cells but not in non-malignant cells. Successful targets of ADC include HER-2, TROP-2 for solid tumors and CD30 and B-cell maturation antigen (BCMA) for hematological malignancies [66]: the former is expressed to some degree in healthy tissues, but are often overexpressed by tumor cells, while CD30 and BCMA expression is restricted to some normal lymphocyte subpopulations [66]. Accordingly, limited target-dependent toxicities, such as pulmonary and cardiac toxicities caused by HER2- targeted ADCs, are reported [66].

## 3. Moving from 2D to 3D Cultures to Model Tumor Microenvironment

Cancer research and drug development have long relied upon experiments performed using in vitro culture of cell lines and primary tumor cells grown in 2D, as well as in vivo animal models [67,68,69]. Traditional 2D cultures, i.e., static cultures of cells kept on flat, plastic supports, still represent the most exploited models for in vitro studies. While these culture systems have provided invaluable information on the basic molecular hallmarks of cancer cells, they present severe limitations, since they fail to accurately recapitulate the spatial and functional complexity of tumor microenvironments, and hence to predict the impact of drugs in individual patients [67,68,69]. On the other side, research on living animals raises ethical issues, which have been addressed through the 3Rs framework (Replacement, Reduction, Refinement) to minimize animal use [70]. Beside ethical concerns, animal models are costly, time-consuming, and require skilled and trained operators to perform specific and reproducible experiments [71]. Finally, these models seem inadequate to faithfully reproduce the complex tumor/stroma interplay occurring in human tumors, thus limiting translation of results to humans [71,72].

Tumors develop within a complex ecosystem made up by several different cellular elements (including mesenchymal stromal cells -MSC-, endothelial cells and immune cells) and organ-specific extracellular matrix (ECM) components that play a fundamental role in supporting tumor cell growth, survival and drug resistance [32]. Thus, experimental cancer models should incorporate elements of the surrounding milieu, encompassing tissue-specific multiple cellularity and architecture, biochemical and mechanical cues, cell–cell and cell-ECM interactions and particularly the three-dimensionality [67,68,69]. Indeed, since the landmark work of Bissell and colleagues [73], several groups have extensively demonstrated that both normal and tumor cells maintained in traditional 2D culture significantly differ from those kept in 3D culture in terms of morphology, biological behavior, gene expression profile and drug sensitivity [74,75]. Thanks to ongoing advancements in tissue engineering and regenerative medicine, 3D platforms have been generated attempting to overcome the limitations of conventional culture models [67,68,69]. These platforms are based on different approaches, also depending on the model’s purpose [76], and can be classified as scaffold-free, scaffold-based, and system-based models [77].

### 3.1. Scaffold-Free Models

**Figure 2:**
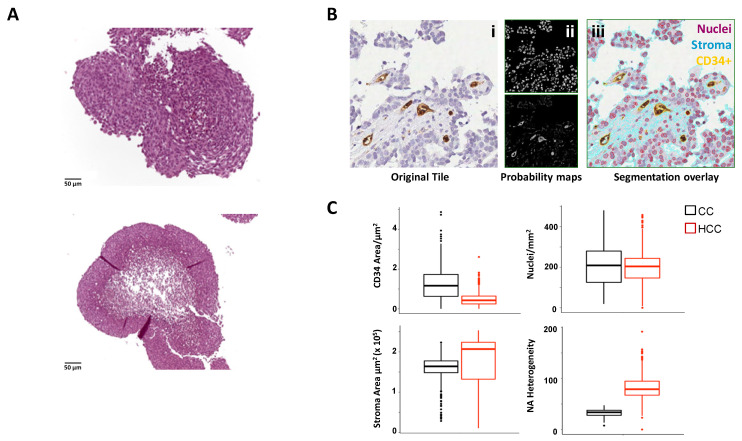
3D culture in RCCS bioreactor of isolated cells and tissues: (**A**) RCCS culture promotes the formation of multilayered spheroids of mesothelioma (AB1 murine cell line) (upper) that develop a necrotic core overtime (lower), thus recapitulating a hallmark of solid tumors. Bars = 50 µm. (**B**,**C**) detection and quantification of tumor angiogenesis in dynamic 3D culture in RCCS bioreactor of cancer tissues; (**B**) machine learning-aided image analysis of ex vivo tissue culture of primary tumor samples (hepatocarcinoma, HCC) allows precise and detailed segmentation of nuclei (haematoxylin), tissue stroma (faint haematoxylin) and CD34^+^ angiogenic vasculature and their unbiased quantification [82]. Original images (i) and pixel-level probability maps (Weka segmentator, ImageJ, (ii) were analyzed with a dedicated Cell Profiler pipeline; (iii) shows structures overlay. In (**C**), whole slides quantifications of CD34^+^ vessels, stroma area, average nuclei area (NA) and heterogeneity of NA in cholangiocarcinoma (CC, black) and HCC (red) samples.

Scaffold-free models include spheroids and organoids [77,78]. The former are clusters of cells that aggregate in suspension, with or without the support of ECM. They can be generated by means of hanging drop techniques or culture in ultra-low adherent plates or in microfluidic systems/bioreactors, taking advantage of the ability of cultured tumor cells to self-aggregate [77,78]. Spheroids derived from tumor cell lines or isolated primary tumor cells, commonly defined tumorspheres, are typically monocultures (homotypic), and therefore poorly recapitulate the complexity and heterogeneity characterizing native tumors [77,78,79]. On the other hand, they are able to mimic the main features of human solid tumors, particularly their structural organization, cellular layered assembling, with a necrotic core surrounded by quiescent cells and a peripheral layer of proliferative cells (Figure 2A), and hypoxia and nutrient gradients. These properties imprint to spheroids an anticancer therapeutics resistance profile similar to that displayed by the parental tumors and are therefore suitable to assess response to chemotherapy [80]. More recently, spheroid models have also been used for the testing of immunotherapies [80,81].

Organoids are self-organizing cell aggregates created by directed differentiation of pluripotent stem cells (PSC) in 3D cultures which differentiate in vitro reproducing the multicellular complexity of the parental organ [82]. These structures are currently being exploited for drug testing, given their suitability for high throughput screenings. The organoid technology has been rapidly adapted to cancer modeling using cells derived from patients’ tumor tissues (tumoroids). The 3D tumoroids more faithfully recapitulate the composition and structure of the tumor they originate from than 2D cultures, thus representing an advancement toward personalized medicine [82,83,84].

Organoids are self-organizing cell aggregates created by directed differentiation of pluripotent stem cells (PSC) in 3D cultures which differentiate in vitro reproducing the multicellular complexity of the parental organ [82]. These structures are currently being exploited for drug testing, given their suitability for high throughput screenings. The organoid technology has been rapidly adapted to cancer modeling using cells derived from patients’ tumor tissues (tumoroids). The 3D tumoroids more faithfully recapitulate the composition and structure of the tumor they originate from than 2D cultures, thus representing an advancement toward personalized medicine [82,83,84].

### 3.2. Scaffold-Based-Models

Additional experimental approaches rely on the use of polymeric substrates with tunable composition and stiffness, as solid scaffolds or hydrogel-based models. Scaffolds are key elements for the generation of 3D platforms, since they provide both the mechanical support and the proper physical and chemical features required for seeded cells to attach, grow and maintain their specialized functions. A suitable scaffold must have favorable biocompatibility or cytocompatibility and adequate pore size and interconnectivity in order to guarantee the growth, differentiation and proper infiltration and distribution of different cell types [83]. Hydrogels are meant to mimic the ECM, and can be either natural or synthetic, the former commonly made with natural polymers (fibrinogen, hyaluronic acid, collagen, Matrigel and gelatin). Synthetic hydrogels are instead typically made with synthetic polymers (polyethylene glycol, polylactic acid, or poly-vinyl acetate) [76].

### 3.3. System-Based Models

System-based models have been developed to overcome static culture conditions, which fail to reproduce the dynamic events occurring in a developing tumor and provide limited mass transfer, that is, gas/nutrient supply and waste elimination, all essential factors for preserving cell viability within large 3D cell/tissue masses [84]. These requirements were addressed using dynamic bioreactors, with the microgravity-based Rotary Cell Culture System (RCCS) bioreactor providing optimal conditions for long-term culture of functional 3D tissue-like bio-constructs and explants of various origin [84,85,86] (Figure 2A). Microfluidic 3D cell culture represents an optimal strategy to generate complex cancer microenvironments and investigate cancer dynamics. The technology allows studying hallmark cellular processes such as cancer proliferation, angiogenesis, migration and invasion, as well as drug responses in a miniaturized, yet well-defined and biologically relevant culture environment [87].

Finally, the 3D bioprinting technology is emerging as an extremely promising strategy, given its potential of manufacturing tissue-engineered compounds with well-defined 3D geometry [88]. In particular, the technique can be used to build tumor constructs via the precise injection of living cells (both tumor and stroma) in functionalized biomaterials (bioinks), thus enabling the spatial–temporal control of molecular, physical and chemical gradients [88,89]. The technology has been recently exploited to print Chronic Lymphocytic Leukemia (CLL) cells (both primary cells and cell lines) mixed with an appropriate hydrogel, thus establishing a reliable and reproducible long-term 3D culture model for leukemia that can be applied to drug testing in a patient-specific context [90].

Particularly demanding is the development of 3D tissue engineered blood vessels, due to their multi-layered cellular composition (endothelial cells, smooth muscle cells, and pericytes), which varies depending on vessel location, lumen size and function; exposure to flow and shear conditions, and expression of vascular tissue functions, including vasoactivity, permeability and secretory functions [91]. Several vascular 3D models are in development, as recently reviewed in [91], ranging from simpler scaffold-free and scaffold-based 3D models [92], to bioprinting techniques, dynamic culture in bioreactors and microfluidic systems. An additional challenge to these approaches is posed by tumor-associated vessels, which, as discussed above, are abnormal, making even more difficult to model a reliable TME suitable to assess the impact of anti-angiogenic therapies.

## 4. Exploiting 3D Models for Therapeutic mAb Testing

The above-described 3D models have been extensively exploited in oncology for drug testing in the perspective of drug screening, precision medicine and personalized treatment of patients. Here we summarize data obtained with the use of 3D models to test therapeutic mAbs, highlighting advantages and potential application together with current limitations (Table 1).

Spheroid models for therapeutic mAb testing include simple approaches, such as the follicular lymphoma model obtained through the hanging drop technique, whereby Vidal-Crespo et al. demonstrated penetration and diffusion of the anti-CD38 mAb daratumumab, as well the resulting spheroid shrinkage, by Selective Plane Illumination Microscopy [93]. Spheroid models have been further implemented by the incorporation of immune cells; in particular, a 3D spheroid co-culture system comprising key components of the diffuse large cell B cell lymphoma (DLBCL) TME, including fibroblasts, myeloid cells and tumor cells, embedded in a collagen-based 3D ECM was generated, with the aim of recapitulating a DLBCL features and to assess immune effector potential using therapeutic mAbs, specifically rituximab for antibody-directed phagocytosis [94].

Courau et al. developed a cancer spheroid model of co-culture of CRC cell lines and immune cells showing activation and induction of tumor cell apoptosis by T and NK cells expressing the NK-like Receptor NKGD2, key activator of cytotoxicity. Moreover, the model allowed testing of the therapeutic potential of mAbs targeting the NKG2D/MICA-B axis, which resulted in increased tumor cytotoxicity by infiltrating immune cells [95]. Varesano et al. determined the anti-tumoral efficacy of Vγ9Vδ2^+^ γδ-T lymphocytes against CRC spheroids by measuring physical characteristics (volume and area) of spheroids as well as their viability. In particular, the authors showed the capability of Vγ9Vδ2^+^ γδ T lymphocytes to mediate ADCC of CRC spheroid subtypes upon cetuximab administration [96]. The same group recently reported the set-up of new 3D culture systems of Hodgkin Lymphoma (HL), i.e., spheroids or collagen scaffolds populated with lymph node (LN) MSC and HL cells. The models allowed to assess the anti-lymphoma effects of the anti-CD30 ADC brentuximab-vedotin and to verify the synergistic effect of ADAM-10 inhibitors, which increase CD30 surface expression in tumor cells by preventing its shedding [97]. Spheroids generation is easy and relatively inexpensive, thus being suitable for high-throughput technologies; however, the technique fails to recapitulate the complex heterogeneity of the parental TME. Moreover, production of uniformly sized spheroids, pre-requisite to readouts comparison and reliability, is still challenging, prompting the development of dedicated technologies that allow their physical characterization, sorting and recovery [98].

Given the urgent need to predict response to IC blockade in individual patients and the restricted availability of reliable biomarkers for preclinical evaluation of immunotherapy, substantial efforts are dedicated to the development of 3D models capable of reproducing tumor/immune cell interactions in a patient specific TME context. Ideally, in vitro cancer cultures should derive from the tumor site and contain critical components of the TME, specifically tumor infiltrating immune cells, thus avoiding an artificial reconstitution. Along this line, murine and patient-derived organotypic spheroids were employed to model short-term responses to PD-1/PD-L1–targeted therapies ex vivo using a novel microfluidic culture system. The model allowed to assess cytokine expression together with anti–PD-1–mediated T-cell activation and subsequent tumor cell death by fluorescence cell imaging and to explore the efficacy of a combination therapy with cyclin-dependent kinase (CDK)4/6 inhibitors [99,100].

A further advancement in the development of 3D tumor microenvironments particularly suitable to modelling immunotherapy with IC blockade was reported by Neal et al. [101]. In this model, the interactions of tumor cells with autologous tumor infiltrating lymphocytes (TIL) within the tumor stromal and immune TME have been successfully recreated through the generation of patient-derived organoids (PDO) from tumors of different histotypes kept in culture in an air-liquid interface [101]. Notably, PDO retained both the parental genetic tumor abnormalities and the native intra-tumoral T cell receptor repertoire of CD3^+^ TIL and allowed to evaluate the enhancement of their tumor-specific cytotoxic function upon IC blockade with the anti-PD-1 antibody nivolumab [101]. Possible limitations to the use of organoids in predicting patient-specific response to drugs rely in the variable success rate and by prolonged time (several weeks) required to obtain and expand the structures.

Hydrogel-based models have also been adopted to test the impact of bsAbs. A novel bsAb, linking the tumor-reactive Vγ9Vδ2 T Cell Receptor (TCR) to a CD3-binding moiety, has been tested in a 3D bone marrow niche model, consisting of stromal cells, endothelial cells and MM cell lines or primary CD138^+^ MM cells embedded in a matrigel layer. Infiltration of immune cells and selective eradication of primary MM cells could be documented in the presence of the bsAb by confocal microscopy [102].

The 3D models in bioreactors have been developed to perform long-term, dynamic cultures of tissues or constructs made of isolated cells in scaffolds/hydrogels. We exploited the dynamic RCCS bioreactor to culture tumor tissues of various origin that exhibited well preserved tissue architecture and cell viability; moreover, specialized functions and metabolic rewiring of both tumor and TME could be assessed in culture supernatants [105,106]. The system was suitable to investigate the impact of drugs, including therapeutic mAbs. Specifically, 3D cultures of tissue biopsies derived from patients affected by Erdheim Chester Disease (ECD), a rare inflammatory myeloid neoplasm driven by MAPK mutations (particularly the BRAF^V600E^ mutation) [107,108], allowed to unveil immune-metabolic mechanisms underlying disease pathogenesis and to compare the efficacy of IFX versus the specific BRAF-inhibitor vemurafenib in dampening inflammation [103]. Culture in bioreactor of fresh tissue explants (organotypic culture) allows assessment of the dynamic evolution of tumor angiogenesis through serial sampling followed by histological and image analysis of tumor features (e.g., nuclei, stroma and angiogenic CD34^+^ vessels). These features can be automatically assessed and quantified via High Content Image Analyses (HCIA) [82], as exemplified with HCC and cholangiocarcinoma (CC) explants (Figure 2B,C). These data represent a proof-of-concept to the use of the RCCS system to predict the efficacy and estimate optimal dosage/timing of anti-angiogenic drugs as normalizing agents, including bevacizumab and its derivatives in a patient-specific context. Limitations to the use of 3D tissue culture models in bioreactor are represented by the availability of tumor samples from surgery, elevated costs and relatively low throughput.

The bioreactor technology was also exploited to model 3D surrogate bone marrow microenvironments of MM and Chronic Lymphocytic Leukemia through co-culture of isolated tumor cells and stroma in scaffolds [109,110]. The resulting constructs maintained long-term tumor cells viability and reproduced proper and functional tumor-stroma interactions, allowing the testing of the efficacy of currently used therapeutics on both compartments [109,110]; the model can be in principle applied to therapeutic mAbs.

An alternative approach relied on a novel flat 3D cell culture bioreactor, named VITVO, which can be loaded with tumor and/or normal cells over a hydrophilic matrix, allowing in vitro reconstruction of tissue complexity. The biocompatible device supported 3D growth of tumor cell lines and enabled investigation of the anti-cancer potential of chemotherapy, biologic agents and cell-based therapy in 3D cytotoxicity assays. Notably, the system could be also exploited to predict responsiveness to the ICI nivolumab using primary tumor cells harvested from lung cancer patients [104].

Of note, all 3D models addressing direct or antibody-mediated cytotoxicity of tumor cells should consider and incorporate the issue of effector cells infiltration in complex systems, which was shown to be critical in testing the cytotoxic potential of immune cells [111] and depends on biomaterials and on culture technology. So far, both natural hydrogels, including matrigel and hyaluronic acid [102,112,113] and spheroids/organoids [95,96,99,100,101] were shown to allow a proper penetration of immune effectors.

Additional preclinical models reproducing human immune-tumor interactions, including bio-printing and microfluidic platforms, while still scarcely exploited in the field of tumor immunology, remain promising candidates in the development of immunotherapies and combination therapies [114,115,116], conceivably providing information not only on therapeutic mAbs efficacy, but also on potential unwanted adverse effects [116].

## 5. Conclusions

3D models are emerging as an invaluable tool for drug development and the design of patient-specific therapeutic interventions in clinical oncology. This is particularly true for therapeutic mAbs, which have already revolutionized personalized cancer therapy and hold promise for further future applications. The use of 3D models is intended to bridge the gap between conventional in vitro cultures and in vivo animal models, overcoming their intrinsic limitations, and to accelerate transition from existing preclinical screening methods to clinical studies. Indeed, several promising 3D models are already available for the investigation of cancer biology and drug testing; however, they still face several drawbacks, including restricted availability of tailored evaluation and quantification methods and insufficient validation, standardization and data analyses tools. Ongoing development of biological sciences, coupled with dedicated biotechnologies allowing specific and easy-to-handle analyses, and of biomaterials closely mimicking tumor graded ECM components and physico-chemical and mechanical structure, are expected to increasingly approximate the molecular and functional interactions between tumor and its native TME. This in turn may result in a broader application of 3D models in both cancer drug research and development and in preclinical design of personalized therapies.

## Figures and Tables

**Table 1:** 3D models for testing of therapeutic mAbs.

3D models		Advantages	Limitations	Applications	Refs.
Spheroids	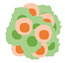	Limited culture requirements Increased cell-cell and cell-matrix interactions Nutrient and oxygen gradients High through-put drug screening Low costs	Mostly monocultures (cell lines) Difficult experimental standardization Uneasy setting of functional assays Complex quantification of response	Flow cytometry Immunohistochemistry Live imaging Immunofluorescence ATP content assay Glucose dosages	[93,94,95,96,97,98]
Patient-derived Organoids	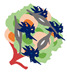	Native tumor heterogeneity Preservation of TME complexity, including TILs High through-put drug screening Biobanking	Variable success rate Time -consuming High costs Need of advanced tools for analysis	Flow cytometry Immunohistochemistry qRT-PCR LIVE/DEAD assay Cytokine detection Single Cell Gene Enrichment Analysis Immunofluorescence	[99,100,101]
Hydrogels	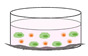	Easy to handle Minimal culture requirements Easy drug testing and experimental standardization Low costs	Lack of TME complexity Limited architectural organization	Flow cytometry Confocal microscopy Cytokine detection	[102]
3D culture inbioreactor	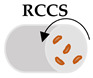	Patient specificity Native tumor and TME Assessment of tumor/TME functions and metabolism Drug testing	High costs Specific expertise required No high-throughput Need of advanced tools for analysis Complex experimental standardization	Confocal microscopy Cytokine detection Immunohistochemistry Glucose/lactate dosages Metabolomics	[103]
	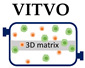	Easy to handle Patient specificity Drug testing	Lack of TME complexity Limited architectural organization	Cell Viability Assay LIVE/DEAD assay	[104]

Abbreviations: TME = tumor microenvironment; TIL = tumor infiltrating lymphocytes. Legend: 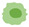 Tumor cell; 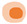 T cell; 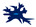 Dendritic cell; 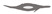 Stromal cell; 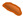 Tissue sample; 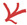 Vessel.

## Data Availability

No new data were created or analyzed in this study. Data sharing is not applicable to this article.
